# Estimating the Illumination Direction From Three-Dimensional Texture of Brownian Surfaces

**DOI:** 10.1177/2041669517701947

**Published:** 2017-04-13

**Authors:** Sylvia C. Pont, Andrea J. van Doorn, Jan J. Koenderink

**Affiliations:** Perceptual Intelligence (π-)lab, Department of Industrial Design Engineering, Delft University of Technology, The Netherlands; Laboratory of Experimental Psychology, University of Leuven, Belgium; Laboratory of Experimental Psychology, Faculty of Social Sciences, Utrecht University, The Netherlands

**Keywords:** Brownian surface, illumination direction, illuminance flow, light field, shading, shadowing, texture

## Abstract

We studied whether human observers can estimate the illumination direction from *3D textures* of random Brownian surfaces, containing undulations over a range of scales. The locally Lambertian surfaces were illuminated with a collimated beam from random directions. The surfaces had a uniform albedo and thus texture appeared only through shading and shadowing. The data confirm earlier results with Gaussian surfaces, containing undulations of a single scale. Observers were able to accurately estimate the source azimuth. If shading dominated the images, the observers committed 180° errors. If cast shadows were present, they resolved this convex-concave-ambiguity almost completely. Thus, observers relied on second-order statistics in the shading regime and used an unidentified first-order cue in the shadow regime. The source elevations could also be estimated, which can be explained by the observers’ exploitation of the statistical homogeneity of the stimulus set. The fraction of the surface that is in shadow and the median intensity are likely cues for these elevation estimates.

## Introduction

We will consider texture due to the illumination of rough surfaces. The appearance of such three-dimensional (3D) textures is dependent on the illumination and on the viewing direction and can be characterized by bidirectional texture functions or BTFs (Curet, 1997; Dana, van Ginneken, Nayar, & Koenderink, 1997, 1999). Conversely, the texture appearance might provide us with cues about the illumination and viewing directions. Luminance distribution or histogram-based cues are, for instance, the width, average, and skewness of the luminance distribution (Ho, Landy, & Maloney, 2008; Kim & Anderson, 2010; Motoyoshi, Nishida, Sharan, & Adelson, 2007; Pont & Koenderink, 2005, 2008; Wijntjes & Pont, 2010). In addition to such relatively easy derivable cues, the spatial properties of 3D textures also provide cues about the illumination, material, and shape (Chantler, Schmidt, Petrou, & McGunnigle, 2002; Gerhard & Maloney, 2010; Karlsson, Pont, & Koenderink, 2008, 2009; Kim, Marlow, & Anderson, 2014; Knill, 1990; Koenderink, 2012; Pont & Koenderink, 2008; Shepard & Campbell, 1998; Varma & Zisserman, 2004). The second-order statistics of shaded 3D textures provides us with estimates of the azimuth of the average illumination orientation, that is, the direction modulo 180° (Koenderink & Pont, 2003; Koenderink, van Doorn, Kappers, te Pas, & Pont, 2003; Koenderink, van Doorn, & Pont, 2004, 2007). The 180° ambiguity is due to the convex-concave-ambiguity. Furthermore, for arbitrary textures (i.e., statistically inhomogeneous sets of textures), the elevation cannot be estimated due to the bas-relief ambiguity (Belhumeur, Kriegman, & Yuille, 1997).

Illumination direction estimation is an important prerequisite for estimates of the light field, shape from shading, and material judgments. In this article, we investigate how well human observers are able to estimate the illumination direction from 3D textures, in connection to our interest in light field perception. The light field (Gershun, 1939), or plenoptic function (Adelson & Bergen, 1991), is defined as the irradiance as a function of position and direction and might serve as a radiometric framework for perception. Texture provides us with cues which are additional to shading. Note that Lambertian shading (Horn & Brooks, 1989) is dependent on the normal component of the local light vector, while texture due to surface roughness is also dependent on the tangential component of the local light vector. The ensembles of local illumination orientation estimates over rough 3D objects form patterns, the illuminance flow (Pont & Koenderink, 2003, 2004). The illuminance flow depends systematically on the light field and on the shape of the object (Karlsson et al., 2008, 2009; Pont & Koenderink, 2003, 2004, 2008; Pont, van Doorn, Wijntjes, & Koenderink, 2015) and provides cues about the light field and object shape (Koenderink, 2012; Pont & Koenderink, 2008).

**Figure 1.**
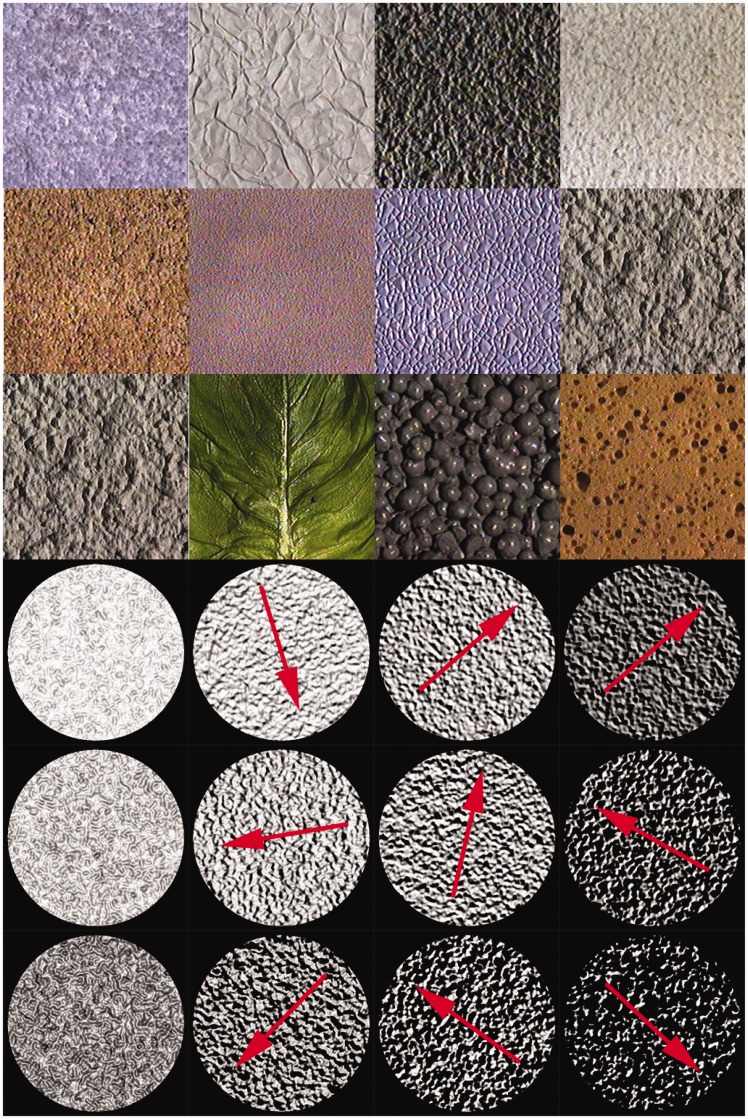
Textures of real materials from the CURET database at the top and rendered textures of Gaussian surfaces at the bottom. All textures were viewed frontally. The Gaussian textures were rendered for a single scale of the roughness, for illumination polar angles of about 0°, 30°, 50°, and 70° (from left to right), varying azimuth (depicted by the red arrows), and for three reliefs, increasing from above to below.

In a previous study, we derived how second-order statistics based on the squared gradient and on the Hessian relate to the illumination direction (Koenderink & Pont, 2003). This relation suggests that the illumination orientation can be derived from 3D textures via responses of so-called *edge and line detectors*. In another study (Koenderink et al., 2003), we tested whether human observers were actually able to carry out this task for frontally viewed real 3D textures from the Curet database, see, for examples, Figure 1 left, and we found that the experimental results were surprisingly similar to the theoretical predictions. The similarity was especially surprising because real textures do not comply with the strict assumptions of the theory at all namely, that the geometry is Gaussian, that the material is locally perfectly matte (Lambertian shading), and that the relief is shallow such that no shadows and no interreflections occur.

In still another study (Koenderink et al., 2004), we carried out a similar experiment for frontally viewed computer-generated Gaussian surfaces, see, for examples, Figure 1 bottom, and again found that human observers’ estimates were close to the fiducial orientation values (interquartile intervals of the deviations of the azimuthal estimates were below 14°). However, for the Gaussian textures in the shadowing regime, we found that observers were able to resolve the convex-concave-ambiguity. The main difference between the textures in the shadowing and shading regimes was the presence of cast shadows. This suggests that observers make use of the difference between the boundaries of the cast shadows and the body shadows, the latter being much more gradual than the former.

Next, we tested illumination direction estimation for textures of frontally viewed Gaussian anisotropic rough surfaces (Koenderink, van Doorn, & Pont, 2007). For such textures, one expects systematic errors of the settings as a function of the anisotropy (Karlsson et al., 2008, 2009). Our expectations were fully borne out, in that the observers committed the predicted systematic errors. The results were precise enough to allow the inference that illumination direction detection is based on second-order statistics, that is, of edge detector (rather than line detector) activity.

Figure 1 shows examples of real materials and of rendered Gaussian surfaces. The Gaussian textures look somewhat artificial and as if photographed out-of-focus. The main reason might be that the surface roughness of these textures is restricted to a single scale, while in natural materials one typically finds undulations over a range of scales (Green, Padilla, Drbohlav, & Chantler, 2007; Kube & Pentland, 1988; Padilla, Drbohlav, Green, Spence, & Chantler, 2008; Wainwright & Simoncelli, 2000). Therefore, in this article, we tested whether a deviation from the theoretical assumption of Gaussian geometry (while the theoretical assumptions of Lambertian reflectance and uniform albedo were fulfilled) will systematically affect the estimates of human observers. We rendered images of height profiles resulting from linear superpositions of a range of Gaussian surfaces of different scales. Due to the effect that larger bumps might put smaller ones in cast shadow, such a “Brownian image texture” is not simply a linear superposition of the image textures of the composing Gaussian surfaces. Can human observers estimate the illumination orientation for these, more realistic surface profiles, containing roughness at a range of scales?

## Methods

### Stimuli

**Figure 2.**
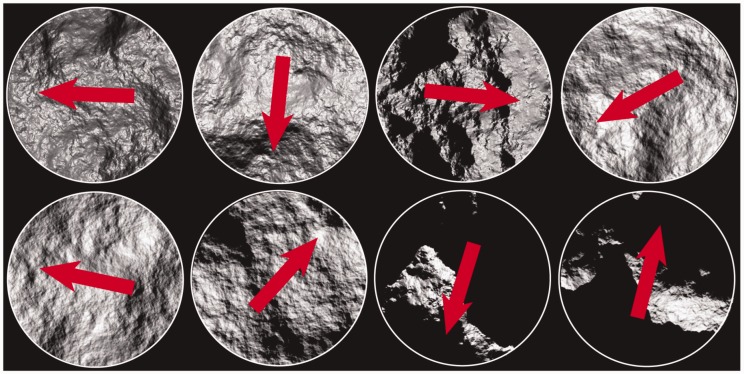
Examples of the stimuli, with arrows depicting the true illumination direction. The cut-out was circular in order to prevent biased responses due to oriented contours.

**Figure 3.**
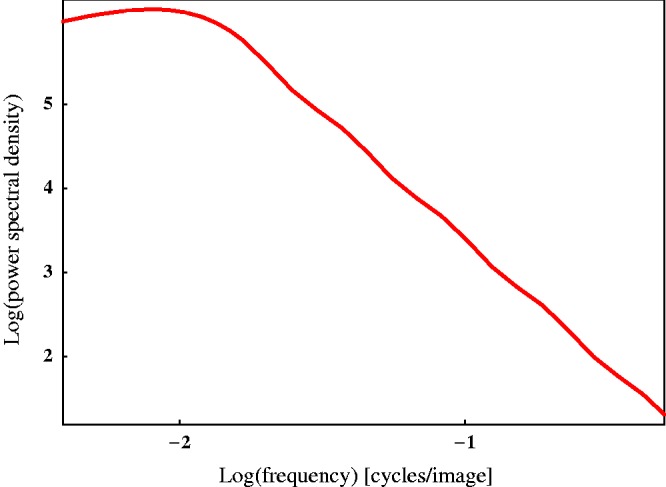
A depiction of the powerspectrum of the *surface height profiles* of our stimuli (log power spectrum density as a function of frequency).

We generated 250 images of frontally viewed Brownian surfaces, see Figure 2 for examples. To create these images, we first constructed statistically independent random surfaces (i.e., surface height profiles) for each of them. The surfaces were generated by linear superposition of seven random Gaussian reliefs of different scales. Each Gaussian relief component was generated with normally distributed heights and an isotropic Gaussian correlation function (Longuet-Higgins, 1957). Hereafter, we set *scale* as the half-width of the autocorrelation function. The scales of the seven Gaussian relief components were increased exponentially to 1, 2.2, 5.0, 11.3, 25.4, 57.0, and 128 pixels, in other words as 0.2%, 0.4%, 1.0%, 2.2%, 5.0%, 11.1%, and 25% of the image-width. The root mean square spreads in the heights were taken proportional to the scales. After superposition of the seven Gaussian random relief components, we subtracted the linear trends of the generated height profiles in order to avoid global slants of the surfaces with respect to the fronto-parallel surface. Finally, the variance of the surfaces was normalized, and the height scaled with a constant of 128. Thus the surface structures had a fractal like character, see the powerspectrum in Figure 3. Because of this fractal like character we called them *Brownian surfaces*.

The stimuli were prepared through a *Mathematica* program and saved as grayscale TIFF-formatted image files of 512 × 512 pixels, 8 bit per pixel, linearly mapping the luminance values. The stimuli were rendered assuming locally Lambertian or perfectly diffuse scattering (Lambert, 1760), a collimated beam (similar to direct sunlight), with pixels in body and cast shadows being set to black (no *ambient term*). These stimuli represent physically realistic renderings, except for the fact that multiple scattering is not present. Note that, although the surface structure or height profile is simply a linear superposition of the surface structures of the composing Gaussian surfaces, the resulting image structure or 3D texture is not a simple combination. This is because the shadows of larger bumps might overcast smaller bumps and in effect make smaller bumps invisible.

**Figure 4.**
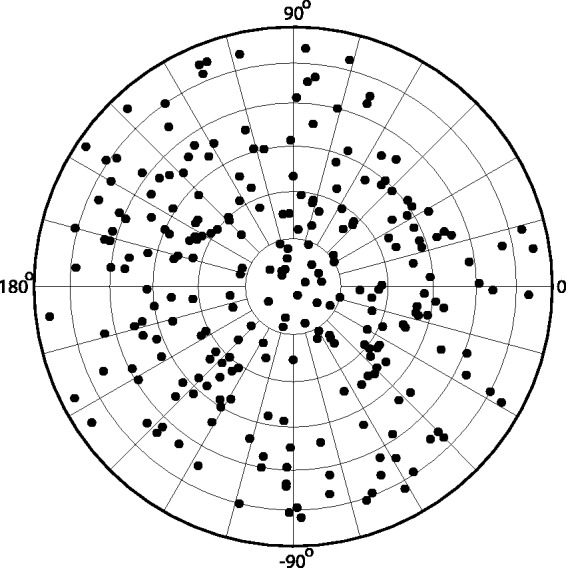
The parameters of the 250 stimuli. The grid specifies 15° increments in azimuth and elevation, using equal-area projection. The convention for the specification of the azimuth (zero direction toward the right, increase in counter-clockwise direction) is used throughout the article. Elevation is measured by the polar angle, that is, the distance to the direction of normal incidence (at the center of the graph). The elevation and azimuth specify the direction toward the light source.

The illumination directions of the 250 images were distributed randomly over the hemisphere of potential illumination directions (see the polar plot in Figure 4). All stimuli were presented in randomized order. The rendered images were shown in a circular mask in order to avoid a possible bias due to the square shape of the images. The test was conducted using a linearized monitor (unit gamma; implemented via software and checked with a gray scale and Koninca Minolta luminance meter).

### Observers

Six observers, the authors and three naive observers, participated in the experiment. Authors S. P. and A. D. were naive with respect to the stimulus parameters. All six observers had normal or corrected-to-normal vision. The experiment was done in accordance with local ethical guidelines, Dutch Law, and with the Declaration of Helsinki.

### Experimental Setup

The setup consisted of an Apple Macintosh G4 and a luminance linearized, 22″ LaCie Blue Electron monitor, at 75 Hz and 1600 × 1200 resolution. Participants were seated with their heads in a chinrest, 83 cm from the screen. Vision was binocular, and the head was fixed through the chin rest. The stimulus and probe (see next subsection) images extended visual angles of 8.6° × 8.6° each. The room was dark during the course of the experiment.

### Design and Procedure

**Figure 5.**
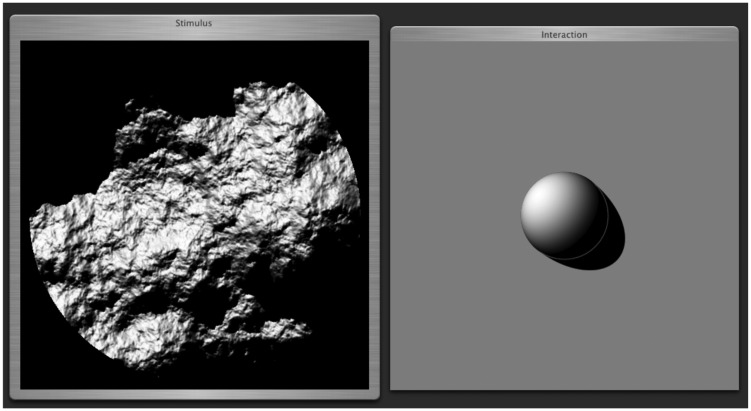
The interface with a stimulus (left) and interaction panel (right). The observers could adjust the direction of the virtual light source that determined the rendering in the response panel. This rendering served to indicate both the elevation and the azimuth of the illumination.

We defined a “natural” interface in the form of a monochrome rendering of an illuminated hemispherical boss on a plane (see Figure 5). The boss and plane were rendered using (Lambertian) shading, with body and cast shadows, without reflexes. The observer could use the mouse in order to adjust the direction of the (simulated) source. The task was to let the illumination of the hemispherical boss appears the same as the illumination of the texture. This proved indeed to be an intuitive interface to all observers in our former and present studies. The median time for a judgment was less than 8 seconds.

## Results

**Figure 6.**
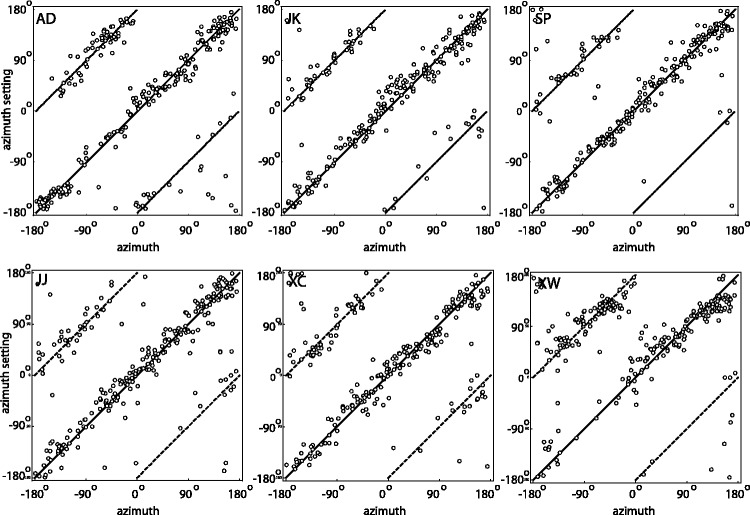
Scatter plots of the azimuth settings (vertical axes) against the ground truth (horizontal axes) for each observer. The drawn lines depict the lines of expectation (ground truth modulo 180°).

**Figure 7.**
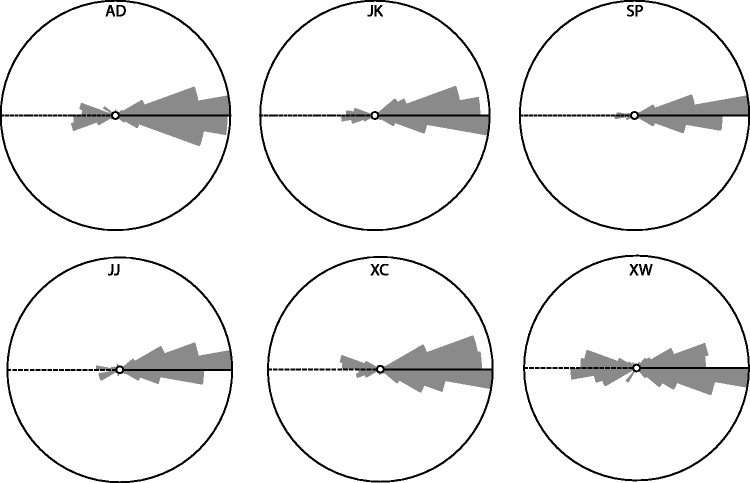
Polar histograms of the azimuthal errors per observer (thus each plot totals 250 trials). Notice that the veridical direction is toward the right.

**Figure 8.**
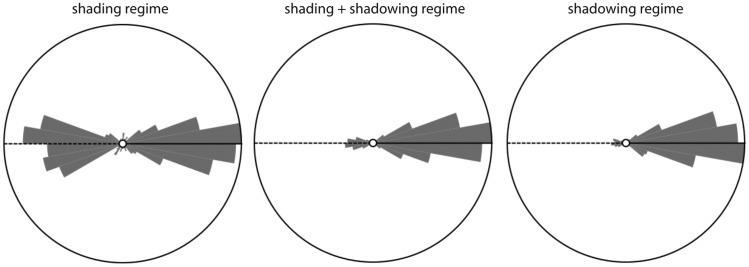
Polar histograms of the azimuthal errors committed by all observers, split into three regimes: polar angles smaller than 30° (the shading regime, left plot, and based on 402 settings), between 30° and 60° (both shading and shadowing happen, middle plot, and based on 750 settings), and larger than 60° (shadowing dominates, right plot, and based on 348 settings).

Figure 6 shows the settings (dots) of the azimuthal angles against the stimulus azimuths, per observer. The drawn lines represent the veridical values modulo 180°. Surprisingly, most settings seem to lie close to the true values, while one would expect about half of them to be 180° off due to the convex-concave-ambiguity. Figure 7 shows the polar histograms of the deviations of the azimuthal settings from the actual illumination azimuths, for all six subjects. Obviously, the number of deviations near 0° is different from the number of deviations that are 180° off, and clearly outnumbers it, confirming that most responses were clustered around the fiducial illumination orientation. Since this result contradicts naive expectations, we did some further analysis on these data. Figure 8 splits the data in three groups for three separate elevation ranges: with a polar angle of 0° to 30° (in which range shading dominates) in the left plot, 30° to 60° (in which range neither shading nor shadowing dominates) in the middle plot, and 60° to 90° (in which range shadowing dominates) in the right plot. It is clear from this figure that in the shading regime indeed (following our expectations), about half of the data is 180° off. However, in the intermediate and in the shadowing regimes, almost all settings are close to the veridical illumination orientations. We calculated the ratios of the numbers of datapoints in the first plus fourth quadrant with respect to the stimulus values to those in the second plus third quadrant. We found that the ratios in the shading, mixed, and shadow regimes were 185/217 = 0.85, 156/594 = 0.26, and 41/307 = 0.13. Thus, the presence of shadows in the image seems to resolve the convex-concave-ambiguity.

**Figure 9.**
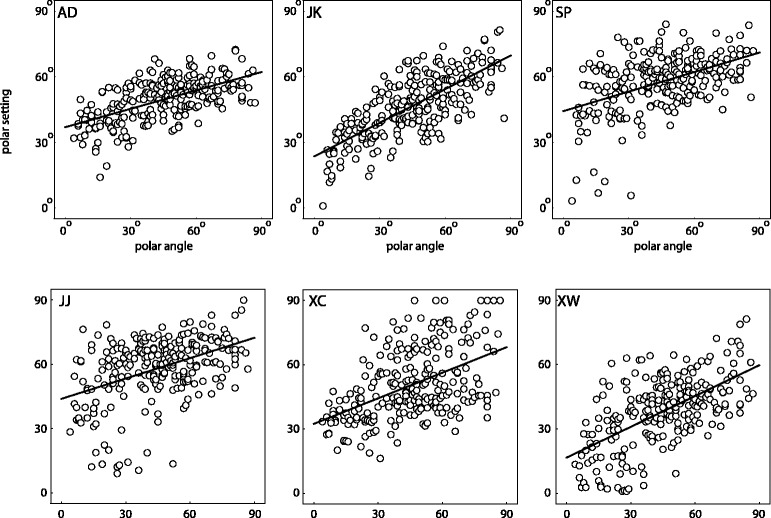
Scatter plots of the polar angle settings (vertical axis) against the ground truth (horizontal axis) for each observer. We fitted the data linearly (drawn lines).

Figure 9 shows the settings (dots) of the polar angles against the stimulus values, per observer. Theoretically, the elevation cannot be estimated due to the bas-relief ambiguity (Belhumeur et al., 1997), so here we expected no clear relation of the settings with the veridical values. Because the data seem to show some correlation with the stimulus values we did a regression on the data. The lines represent linear fits to the veridical polar angle θ, for which we found:

37°+ 0.28 θ with *R*^2^= 0.35 for A. D.,

24°+ 0.51 θ with *R*^2^= 0.54 for J. K.,

44°+ 0.30 θ with *R*^2^= 0 .22 for S. P.,

44°+ 0.32 θ with *R*^2^= 0.19 for J. J.,

32°+ 0.40 θ with *R*^2^= 0.24 for X. C., and

17°+ 0.48 θ with *R*^2^= 0.36 for X. W.

**Figure 10.**
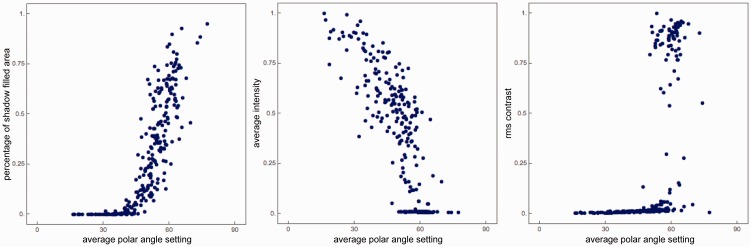
Scatterplots of the average polar angle settings of all observers and the percentage of shadow-filled area (left), the average intensity (center), and the root mean square contrast (right). Notice that shadowing sets in at a polar angle of about 30°, and that the settings correlate nicely with the shadow fraction. The median intensity also acts as cue in the shading regime, not just in the shadow regime.

To understand this slight but significant correlation, we looked at the correlation between the data and a few possible effective cues that the observers might have used. Since the stimulus set is homogeneous in terms of statistics, observers might have used cues such as the average gray level, contrast, shadowed fraction of the total area. Figure 10 shows the average polar angle settings (horizontal axis) of the observers against the shadow fraction, median intensity, and Michelson contrast (from 5% to 95% percentiles instead of the absolute minimum and maximum). It is clear that the settings correlate nicely with the shadow fraction. The median intensity also acts as a cue in the shading regime, not just in the shadow regime. For the contrast, we find a distinct picture. The contrast *explodes* at about 60° due to the dominance of cast shadows of big bumps, which put very large parts of the image in shadow (including small bumps). Even in this large-scale shadow dominated regime, observers were able to conduct our task well.

In addition to the former analysis, we studied (straight) correlations between the observers’ azimuthal data (O) and illumination orientation estimates (E) that were calculated from second-order statistics on the basis of the squared gradient—that is, of edge detector (rather than line detector) activity. Our former studies suggested that such a mechanism might underly illumination orientation detection (Koenderink & Pont, 2003; Koenderink et al., 2003, 2004, 2007). The estimates were calculated for three differentiating scales of our algorithm (1, 8, and 64 pixels) and averaged over the inner square of the stimuli of 344 pixels squared in which there was no coverage by the circular mask. Correlations were computed for the orientations, rather than directions, in other words, we corrected for 180° flips. We also computed the correlations between these illumination orientation estimates (E) and the true azimuths (T), for comparison with the correlations between the illumination orientation estimates and the observers’ azimuthal settings (E–O). Finally, as a sort of baseline correlation, we computed the observers’ azimuthal settings against the true azimuths (O–T). These correlations were computed separately for the shading, intermediate, and shadowing regimes. The results are represented in Table 1.

**Table 1.** Correlation Coefficients for Comparison Between the Illumination Orientation Estimates (E) and the True Azimuths (T), Between the Illumination Orientation Estimates and the Observers’ Azimuthal Settings (E–O), and Between the Observers’ Azimuthal Settings and the True Azimuths (O–T). The illumination orientation estimates were computed at three different scales (second column). These correlations were computed separately for the shading, intermediate, and shadowing regimes (Columns 3–5).

Comparison	Scale (pixels)	Correlation in shading regime	Correlation in intermediate regime	Correlation in shadowing regime
O–T	Not applicable	0.80	0.93	0.88
E–O	1	0.82	0.93	0.88
E–T	1	0.88	0.997	0.995
E–O	8	0.81	0.93	0.91
E–T	8	0.77	0.97	0.97
E–O	64	0.47	0.71	0.60
E–T	64	0.46	0.71	0.60

These numbers confirm that observers could estimate the illumination orientations rather well (O–T correlations are quite high). The correlations of the observers’ settings and of the illumination orientation estimates with the true estimates were consistently higher for the intermediate regime than for the shading or shadowing regimes. Moreover, the correlations for the intermediate regime were most robust under variation of the differentiating scale. The correlations for the shading regime show the largest decrease with increasing scale. The correlations of the estimated illumination orientations at the largest scale (E–O and E–T) were clearly lower than those of the observers (O–T). Summarizing, we find that the second-order statistics correlated well with the observers’ settings, especially at lower scales, and especially in the intermediate regime.

## Conclusions and Discussion

The main conclusion from this study is that a deviation from the theoretical assumption of Gaussian geometry does not affect the estimates of human observers systematically. Human observers can estimate the illumination orientation for our more realistic surface profiles containing roughness at a range of scales. Moreover, the presence of a range of scales, instead of a single scale of the undulations, prevented complaints by the observers. In our previous work on random Gaussian surfaces, we found that observers “did not ‘like’ the samples because they appear somewhat ambiguous” (Koenderink et al., 2004). The stimuli in the current study are probably more pleasant to view because they do look sharp and they do offer a “hold” to the eye (as distinct from the Gaussian surfaces). The results from the current study confirm earlier results using texture images from the CURET database (Curet, 1997). Moreover, as in the case of our study using rendered random Gaussian surfaces (Koenderink et al., 2004), we found that observers were quite capable at elevation and azimuthal direction (instead of orientation) estimation.

The observers’ sensitivity to light source elevation cannot be interpreted as an absolute sensitivity to the height of the light source. Such sensitivity is impossible in view of the *bas-relief ambiguity* (Belhumeur et al., 1997), which is a basic image ambiguity concerning light source elevation and relief height. The reason must be the statistical homogeneity of the stimulus set. Observers might have used, for instance, the average brightness and shadow fraction to grade the samples and relate them to some equivalent elevation scale for the experiment. In contradistinction to our study on Gaussian textures, we did not find a monotonic relation of the settings with contrast, so the contrast cannot serve as a direct cue in the current study.

**Figure 11.**
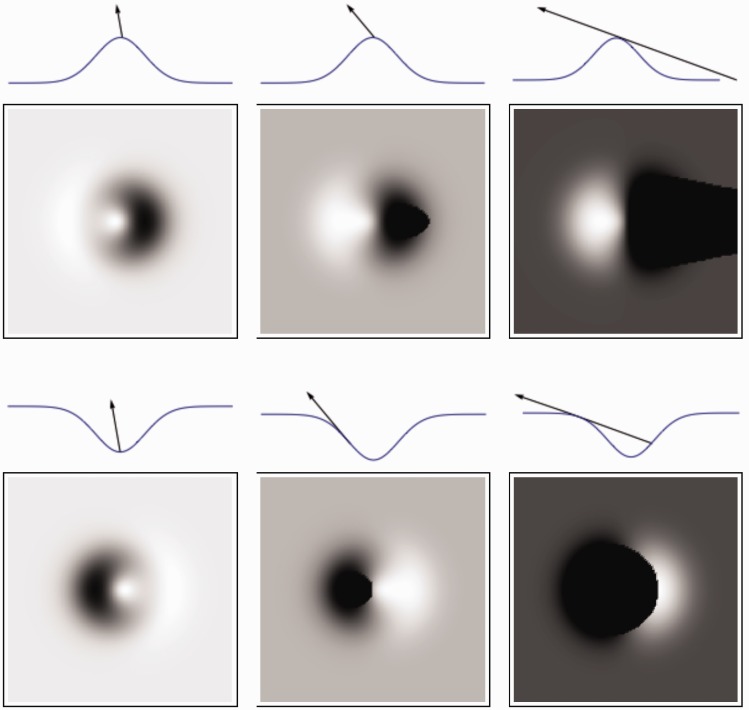
Surface profiles with arrows representing illumination directions (the first row for each set). The second row for each set shows the images which correspond with the illumination directions represented above them. The first set shows images of a bump and the second set of a trough in the shading (left), intermediate (middle), and shadowing (right) regimes.

**Figure 12.**
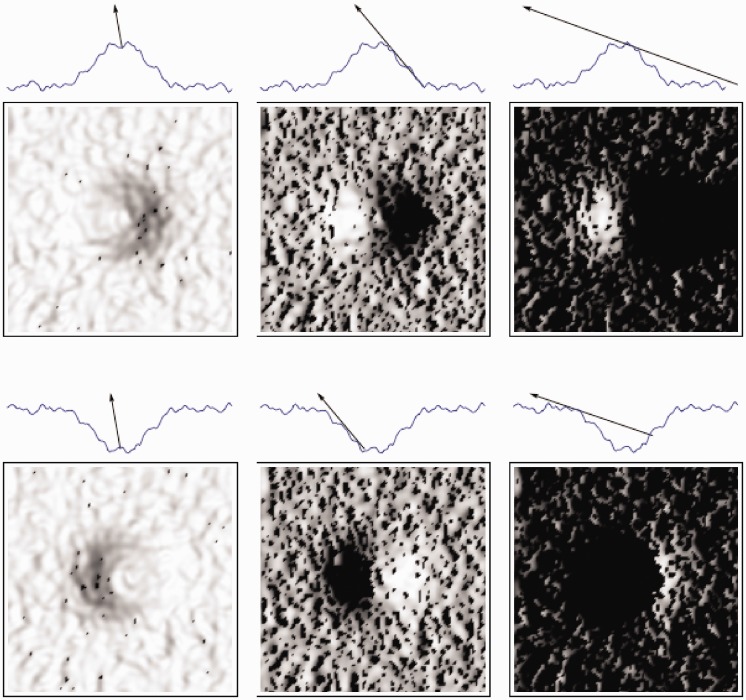
Surface profiles with arrows representing illumination directions (the first row for each set). The second row for each set shows the images which correspond with the illumination directions represented above them. The first set shows images of a bumpy bump and the second set of a bumpy trough in the shading (left), intermediate (middle), and shadowing (right) regimes.

The azimuthal settings in the intermediate and shadowing regimes did not show a 180° modulus. Thus, observers were able to estimate the illumination direction, not just the orientation, if cast shadows were present. Probably the difference between cast and body shadows was used as a cue to the illumination direction, resolving the convexity-concavity-illumination-direction-ambiguity, see Figure 11. Cast shadows have a sharp boundary, while body shadow boundaries are generally more gradual. Noncollimated lighting might thus influence how well the convexity-concavity-illumination-direction-ambiguity direction ambiguity can be resolved because the difference in sharpness of the cast and body shadows might become less clear. The transitions of light-to-dark and dark-to-light in the direction of the tangential component of the light vector are cast shadow edges and body shadow edges. The asymmetric shapes of the shadow and light patches might be another cue for this resolution. In the case of our more natural Brownian surfaces, these differences between cast and body shadows can be much less salient due to the nonlinear combination of such image effects on a range of scales. Figure 12 shows a visualization of how small-scale shadows may *mask* the large-scale cast-body shadow differences in sharpness of the gradients (sharp vs. more gradual boundaries) and the asymmetric shapes of the shadow patches. This masking effect is striking in the intermediate regime of the center images in Figure 12. In addition, in our stimuli, very large-scale cast shadows may put smaller ones in shadow and decrease this masking effect; this effect is very clear in the right images of Figure 12. However, despite these complicating factors—that are omnipresent in most natural materials—observers showed to be quite capable of using the shadowing cues to resolve the 180° ambiguity. It remains to be answered whether the same resolution due to cast shadows occurs in scenes with generic content, that is, objects instead of 3D texture.

In all regimes, we found that the observers’ estimates were accurate in terms of orientation, which suggests that they used shading as well as shadowing cues. The second-order statistics correlated well with the observers’ settings for stimuli in all three regimes, especially at lower scales. The decrease of the correlations for increasing scale suggests that shading and shadowing cues at smaller scales are needed to arrive at the observed accuracy of the observers’ settings and may be combined with large-scale cues—especially in the intermediate and shadowing regimes. It would be interesting to study this point in more detail in combination with eye tracking, to see whether observers look at specific locations in the image. A mechanism combining shading and shadowing cues at a range of scales is of course very convenient with regard to light field estimates in natural scenes.

**Figure 13.**
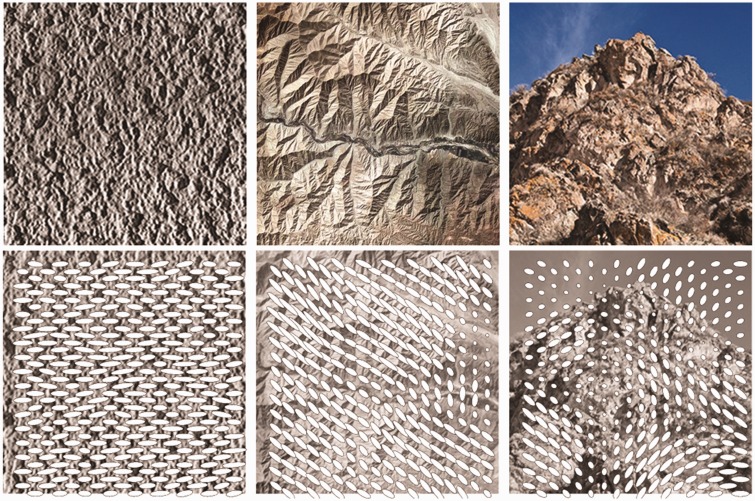
Surface illuminance flow estimates for a flat plaster surface, a mountainous area viewed from above, and a mountain viewed from the side. The ellipsoids’ semimajor axes represent the estimated illuminance flow orientations. The eccentricity represents the confidence level.

Our results show that it is very plausible that ensembles of illuminance flow estimates are an important cue to the light field in natural scenes. Figure 13 shows the computational gradient-based illuminance flow estimates (for the algorithm, see Koenderink et al., 2003) for three photographs: a flat piece of plaster, a mountain area seen from above, and a mountain seen from the ground. The flow estimates are represented by ellipses, with the orientation of the major axis representing the estimated irradiation direction and the eccentricity representing the confidence. On the basis of our findings, we hypothesize that weighted combinations of such flow estimate ensembles at multiple scales are probably an important cue for the visual light field (Koenderink et al., 2007). Recent findings show that the visual light field is simplified in comparison to the physical light field and that observers are sensitive to converging, diverging, and uniform fields (Kartashova, Sekulovski, de Ridder, te Pas, & Pont, 2016; van Doorn, Koenderink, Todd, & Wagemans, 2012), which suggests that such relatively simple topologies might represent templates for (often more complicated) natural light fields. Also, it was shown that scene layout and object properties can influence illumination estimates (Schutt, Baier, & Fleming, 2016; Xia, Pont, & Heynderickx, 2016), which is to be expected if the visual light field is inferred from shading and shadowing patterns. In future studies, we will further study the extrapolation from textures to (perception of) illuminance flow over 3D objects and natural scenes, which is far from trivial due to, for instance, foreshortening and local occlusion effects.

Finally, these observations are of course related to material and shape perception, and not just light. Since we simultaneously infer higher dimensional material, shape, and illumination properties from two-dimensional images, it is to be expected that such inferences interact. Many studies have shown that it is indeed the case that material, shape, and illumination perception is confounded (Gerhard & Maloney, 2010; Ho et al., 2008; Kim et al., 2014; Motoyoshi et al., 2007; Pont & te Pas, 2006; te Pas & Pont, 2005; Wijntjes, Doerschner, Kucukoglu, & Pont, 2012). Looking at our stimuli in the current study, see, for examples, Figure 2, we saw that many of our stimuli did not look as being made of matte material. Many of them tend to look quite glossy or shiny. We studied this illusory gloss in depth in another article (Wijntjes & Pont, 2010), in which we tested gloss perception for Brownian surfaces as a function of the depth range and illumination direction. We found that an interpretation in the context of the bas-relief ambiguity (Belhumeur et al., 1997) could explain our gloss perception data; on average perceived gloss increased with increasing relief and decreased with decreasing source elevation.

Interreflections were ignored in that experiment, as well as in the current experiment. We expect that the addition of interreflections (or an *ambient term*) will not influence our data. We do expect, however, that interreflections rendering will influence the perception of *relief*; a famous observation in this area of study concerns the perceptual overestimation of relief of the moon surface by astronauts (Philips, 2006). Unfortunately, we are still lacking experimental methods to probe surface shape. We belief this is currently one of the biggest challenges to arrive at a more holistic approach in natural material perception. Simultaneous testing of surface relief height, material reflectance, and illumination perception may well be the only manner to fully understand its underlying processes.

## References

[bibr1-2041669517701947] Adelson E. H. Bergen J. R. (1991) The plenoptic function and the elements of early vision. In: Landy M. Movshon J. A. (eds) Computational models of visual processing, Cambridge, MA: MIT Press, pp. 3–20.

[bibr2-2041669517701947] Belhumeur, P., Kriegman, D. J., & Yuille, A. L. (1997). The bas-relief ambiguity. *Proceedings of IEEE Conference on Computer Vision and Pattern Recognition* (pp. 1060–1066). New York, NY: Institute of Electrical and Electronics Engineers.

[bibr3-2041669517701947] Chantler, M., Schmidt, M., Petrou, M., & McGunnigle, G. (2002). The effect of illuminant rotation on texture filters: Lissajous’s ellipses. In A. Heyden, G. Sparr, M. Nielsen, & P. Johansen (Eds.), *ECCV* (LNCS 2352, pp. 289–303). Heidelberg, Germany: Springer.

[bibr4-2041669517701947] Curet (1997). *Columbia-Utrecht reflectance and texture database*. Retrieved from http://www.cs.columbia.edu/CAVE/Curet.

[bibr5-2041669517701947] Dana, K. J., van Ginneken, B., Nayar, S. K., & Koenderink, J. J. (1997). Reflectance and texture of real-world surfaces. *Proceedings of IEEE Conference on Computer Vision and Pattern Recognition* (pp. 151–157). New York, NY: Institute of Electrical and Electronics Engineering.

[bibr6-2041669517701947] Dana K. J. van Ginneken B. Nayar S. K. Koenderink J. J. (1999) Reflectance and texture of real-world surfaces. ACM Transactions on Graphics 18: 1–34.

[bibr7-2041669517701947] Gerhard H. E. Maloney L. T. (2010) Estimating changes in lighting direction in binocularly viewed three-dimensional scenes. Journal of Vision 10: 14. 10.1167/10.9.14 PMC4462141 21106676

[bibr8-2041669517701947] Gershun A. (1939) The light field (P. Moon & G. Timoshenko, Trans.). Journal of Mathematical Physics 18: 51–151.

[bibr9-2041669517701947] Green P. R. Padilla S. Drbohlav O. Chantler M. (2007) Perceived roughness of textured surfaces. Perception 36: 305. 10.1016/j.visres.2008.05.015 18603278

[bibr10-2041669517701947] Ho Y. X. Landy M. S. Maloney L. T. (2008) Conjoint measurement of gloss and surface texture. Psychological Science 19: 196–204. 18271869 10.1111/j.1467-9280.2008.02067.x PMC2679902

[bibr11-2041669517701947] Horn B. K. P. Brooks M. J. (1989) Shape from shading, Cambridge, MA: MIT Press.

[bibr12-2041669517701947] Karlsson S. Pont S. C. Koenderink J. J. (2008) Illuminance flow over anisotropic surfaces. Journal of the Optical Society of America A 25: 282–291. 10.1364/josaa.25.000282 18246161

[bibr13-2041669517701947] Karlsson S. Pont S. C. Koenderink J. J. (2009) Illuminance flow over anisotropic surfaces with arbitrary viewpoint. Journal of the Optical Society of America A 26: 1250–1255. 10.1364/josaa.26.001250 19412244

[bibr14-2041669517701947] Kartashova, T., de Ridder, H., te Pas, S. F., Schoemaker, M., & Pont, S. C. (2015). The visual light field in paintings of museum Prinsenhof: Comparing settings in empty space and on objects. In Bernice E. Rogowitz, Thrasyvoulos N. Pappas, & Huib de Ridder (Eds.), *Human vision and electronic imaging XX. Proceedings of SPIE* (Vol. 9394, pp. 1–10). Bellingham, WA: SPIE.

[bibr15-2041669517701947] Kartashova T. Sekulovski D. de Ridder H. te Pas S. F. Pont S. C. (2016) Global structure of the visual light field and its relation to the physical light field. Journal of Vision 16: 9. 10.1167/16.10.9 27548087

[bibr16-2041669517701947] Kim J. Anderson B. L. (2010) Image statistics and the perception of surface gloss and lightness. Journal of Vision 10: 3. 10.1167/10.9.3 20884601

[bibr17-2041669517701947] Kim J. Marlow P. J. Anderson B. L. (2014) Texture-shading flow interactions and perceived reflectance. Journal of Vision 14: 1–19. 10.1167/14.7.1 24891438

[bibr18-2041669517701947] Knill D. C. (1990) Estimating illuminant direction and degree of surface relief. Journal of the Optical Society of America A 7: 759–775. 10.1364/josaa.7.000759 2338597

[bibr19-2041669517701947] Koenderink, J. J. (2012). Shadows of shape. In *Utrecht: De Clootcrans Press*. Retrieved from http://www.gestaltrevision.be/en/resources/clootcrans-press.

[bibr20-2041669517701947] Koenderink J. J. van Doorn A. J. Kappers A. M. L. te Pas S. F. Pont S. C. (2003) Illumination direction from texture shading. Journal of the Optical Society of America A 20: 987–995. 10.1364/josaa.20.000987 12801166

[bibr21-2041669517701947] Koenderink J. J. Pont S. C. (2003) Irradiation direction from texture. Journal of the Optical Society of America A 20: 1875–1882. 10.1364/josaa.20.001875 14570101

[bibr22-2041669517701947] Koenderink J. J. Pont S. C. van Doorn A. J. Kappers A. M. L. Todd J. (2007) The visual light field. Perception 36: 1595–1610. 18265841 10.1068/p5672

[bibr23-2041669517701947] Koenderink J. J. van Doorn A. J. Pont S. C. (2004) Light direction from shad(ow)ed random Gaussian surfaces. Perception 33: 1405–1420. 15729909 10.1068/p5287

[bibr24-2041669517701947] Koenderink J. J. van Doorn A. J. Pont S. C. (2007) Perception of illuminance flow in the case of anisotropic rough surfaces. Perception & Psychophysics 69: 895–903. 18018970 10.3758/bf03193926

[bibr25-2041669517701947] Kube P. Pentland A. (1998) On the imaging of fractal surfaces. IEEE Transactions on Pattern Analysis and Machine Intelligence 10: 704–707.

[bibr26-2041669517701947] Lambert, J. H. (1760). *Photometria, sive de Mensura et gradibus luminis, colorum et umbrÅ*. Augsburg, Germany: Eberhard Klett.

[bibr27-2041669517701947] Longuet-Higgins M. S. (1957) The statistical analysis of a random moving surface. Philosophical Transactions of the Royal Society, Series A 249: 321–364.

[bibr28-2041669517701947] Motoyoshi I. Nishida S. Sharan L. Adelson E. (2007) Image statistics and the perception of surface qualities. Nature 447: 206–209. 17443193 10.1038/nature05724

[bibr29-2041669517701947] Padilla S. Drbohlav O. Green P. R. Spence A. Chantler M. (2008) Perceived roughness of 1/f noise surfaces. Vision Research 48: 2193–2203. 18603278 10.1016/j.visres.2008.05.015

[bibr30-2041669517701947] Philips, T. (2006). *Apollo chronicles: Dark shadows*. Retrieved from http://science.nasa.gov/science-news/science-at-nasa/2006/03jan_moonshadows/.

[bibr31-2041669517701947] Pont, S. C., & Koenderink, J. J. (2003). Illumination flow. In N. Petkov & M. A. Westenberg (Eds.), *Computer analysis of images and patterns*, *proceedings lecture notes in computer Science* (Vol. 2756, pp. 90–97). Berlin, Heidelberg/Germany: Springer.

[bibr32-2041669517701947] Pont, S. C., & Koenderink, J. J. (2004, September). Surface illuminance flow. In Y. Aloimonos & G. Taubin (Eds.), *Second international symposium on 3D data processing, visualization and transmission*. Symposium conducted at the meeting of Thessaloniki, Greece.

[bibr33-2041669517701947] Pont, S. C., & Koenderink, J. J. (2005). Bidirectional texture contrast function. *International Journal of Computer Vision, 62*, 17–34.

[bibr34-2041669517701947] Pont, S. C., & Koenderink, J. J. (2008). Shape, surface roughness, and human perception. In M. Mirmehdi, X. Xie, & J. Suri (Eds.), *Handbook of texture analysis* (pp. 197–222). London, England: Imperial College Press.

[bibr35-2041669517701947] Pont S. C. te Pas S. F. (2006) Material-illumination ambiguities and the perception of solid objects. Perception 35: 1331–1350. 17214380 10.1068/p5440

[bibr36-2041669517701947] Pont, S. C., van Doorn, A. J., Wijntjes, M. W. A., & Koenderink, J. J. (2015). Texture, illumination, and material perception. In B. E. Rogowitz, T. N. Pappas, & H. de Ridder (Eds.), *Human vision and electronic imaging XX, proceedings of SPIE* (Vol. 9394, p.). Bellingham, WA: SPIE.

[bibr37-2041669517701947] Schutt H. H. Baier F. Fleming R. W. (2016) Perception of light source distance from shading patterns. Journal of Vision 16: 9. 10.1167/16.3.9 26868887

[bibr38-2041669517701947] Shepard M. K. Campbell B. A. (1998) Shadows on a planetary surface and implications for photometric rough-ness. Icarus 14: 279–291.

[bibr39-2041669517701947] te Pas, S. F., & Pont, S. C. (2005). Comparison of material and illumination discrimination performance for real rough, real smooth and computer generated smooth spheres. *Proceedings of the 2nd Symposium on Applied Perception in Graphics and Visualization,* A Coruña, Spain, 75–81. New York, NY: ACM SIGGRAPH.

[bibr40-2041669517701947] van Doorn A. J. Koenderink J. J. Todd J. T. Wagemans J. (2012) Awareness of the light field: The case of deformation. i-Perception 3: 467–480. 23145298 10.1068/i0504 PMC3485835

[bibr41-2041669517701947] Varma M. Zisserman A. (2004) Estimating illumination direction from textured images. CVPR 1: 179–186.

[bibr42-2041669517701947] Wainwright, M. J., & Simoncelli, E. P. (2000). Scale mixtures of Gaussians and the statistics of natural images. In S. A. Solla, T. K. Leen, & K.-H. Müller (Eds.), *Advances of neural information processing* (Vol. 12, pp. 855–861). Cambridge, MA: The MIT Press.

[bibr43-2041669517701947] Wijntjes M. W. A. Doerschner K. Kucukoglu G. Pont S. C. (2012) Relative flattening between velvet and matte 3D shapes: Evidence for similar shape-from-shading computations. Journal of Vision 12: 1–11. 10.1167/12.1.2 22214564

[bibr44-2041669517701947] Wijntjes M. W. A. Pont S. C. (2010) Illusory gloss on Lambertian surfaces. Journal of Vision 10: 1–12. 10.1167/10.9.13 21106675

[bibr45-2041669517701947] Xia L. Pont S. C. Heynderickx I. (2016) Effects of scene content and layout on the perceived light direction in 3D spaces. Journal of Vision 16: 14. 10.1167/16.10.14 27548091

